# Has the Clock Drawing Test been left aside with the replacement of analog clocks by smartphones?

**DOI:** 10.1590/1980-5764-DN-2024-0178

**Published:** 2025-03-21

**Authors:** Anna Alice Vidal Bravalhieri Ribera, Tayla Borges Lino, Nathália Oliveira Rodrigues, Juliana Hotta Ansai, Larissa Pires de Andrade, Gustavo Christofoletti

**Affiliations:** 1 Universidade Federal de Mato Grosso do Sul, Faculdade de Medicina, Campo Grande MS, Brazil.; 2 Universidade Federal de São Carlos, Departamento de Gerontologia, Departamento de Fisioterapia, São Carlos SP, Brazil.

**Keywords:** Bibliometrics, Neuropsychological tests, Aged, Dementia, Bibliometria, Teste do Relógio, Testes Neuropsicológicos, Idoso, Demência

## Abstract

The Clock Drawing Test (CDT) is a widely used tool for dementia screening, assessing executive functions by drawing an analog clock. With the rise of smartphones, many individuals have transitioned from their traditional watches to checking hours digitally. This raises questions about whether difficulties in CDT performance are linked to cognitive decline or lack of familiarity with analog clocks. A potential decline in the number of studies using the CDT may reflect concerns regarding this issue.

**Objective:**

To investigate whether the number of studies using CDT has been affected over the years by the predominance of smartphones over analog clocks.

**Methods:**

This bibliometric study analyzed 1,298 articles published in PubMed over the past 30 years (1994–2023). Data included year of publication region and methodological design. The chi-square test was used for statistical analysis. Significance was set at 5%.

**Results:**

The number of studies using CDT has increased over the past 30 years, from 118 (1994–2003) to 405 (2004–2013) and 775 (2014–2023). Most studies were conducted in Europe (37.6%), Asia (27.7%), and North America (21.0%). The primary focus of the studies was diagnosis (82.2%), followed by treatment (11.5%).

**Conclusion:**

The prevalence of smartphones over analog clocks has not affected the number of studies that have used CDT. Although analog clocks are no longer a part of daily life for many people, this trend does not currently pose an obstacle to the use of the CDT.

## RESUMO

O Teste do Desenho do Relógio (TDR) é uma ferramenta amplamente utilizada para triagem de síndromes demenciais, avaliando as funções executivas por meio do desenho de um relógio analógico. Com a expansão do uso de *smartphones*, muitas pessoas deixaram seus relógios tradicionais de lado e começaram a verificar as horas de forma digital. Isso levanta questões sobre se as dificuldades no desempenho do TDR estão relacionadas ao declínio cognitivo ou à falta de familiaridade com relógios analógicos. Uma queda no número de estudos utilizando o TDR pode refletir preocupações relacionadas a essa questão.

**Objetivo:**

Investigar se o número de estudos utilizando o TDR foi afetado ao longo dos anos pelo predomínio de *smartphones* em relação a relógios analógicos.

**Métodos:**

Este estudo bibliométrico analisou 1.298 artigos publicados na base PubMed nos últimos 30 anos (1994–2023). Os estudos foram comparados em termos de ano de publicação, região e desenho metodológico. O teste qui-quadrado foi utilizado nas análises inferenciais. Significância estatística foi definida em 5%.

**Resultados:**

O número de estudos utilizando o TDR aumentou nos últimos 30 anos, passando de 118 artigos (1994–2003) para 405 (2004–2013) e 775 (2014–2023). A maioria dos estudos foi desenvolvida na Europa (37,6%), Ásia (27,7%) e América do Norte (21,0%). O foco principal dos estudos foi diagnóstico (82,2%), seguindo pelo tratamento (11,5%).

**Conclusão:**

O predomínio de *smartphones* sobre relógios analógicos não afetou a quantidade de publicações envolvendo o TDR. Embora os relógios analógicos não façam parte do cotidiano de muitas pessoas, essa tendência até o momento parece não representar um obstáculo ao uso do TDR.

## INTRODUCTION

With the increasing life expectancy of the population, brain disorders are becoming more prevalent and require early diagnosis^1^. Dementia, one of the most common brain disorders associated with aging, is characterized by progressive cognitive decline that affects older adults and their families^2^.

Diagnosing dementia is a complex process that often relies on laboratory and neuroimaging data, which are not always available to community settings^3^. Several tools have been developed to assist health professionals in clinical screening. These tools involve tasks requiring specific skills that enable an understanding of a person’s cognitive functions. Although the instruments do not provide a definitive diagnosis, they are crucial in guiding physicians on their course of action^4,5,6^.

Among the tools utilized to assess cognitive functions and screen for dementia, the Clock Drawing Test (CDT) is one of the most commonly used^7^. The test involves asking a person to draw an analog clock with all its numbers, followed by a request to draw the clock hands indicating a specific time, after which a score was given. The advantage of CDT is that it is easy and quick to administer, and it evaluates multiple cognitive functions, including selective attention, auditory comprehension, verbal working memory, numerical knowledge, memory and visual reconstruction, visuospatial skills, motor execution, and executive functions^7,8^. However, a notable limitation of the CDT is its inapplicability to illiterate individuals^9^.

The essence of the CDT lies in recalling an analog clock and drawing it on paper. Its consistent psychometric proprieties, good inter-rater and test-retest reliability, and ease of application make the CTD a widely available cognitive screening tool in clinical settings for patients suffering from brain injuries, including dementia. Successful performance of the test depends on the patient’s experience with analog clocks and their functional understanding. Individuals with little experience with analog clocks may have difficulty with the task, which does not necessarily indicate dementia^7,8,9,10^.

A global phenomenon that has intensified in recent years may have affected the CDT. This phenomenon began in the mid-1980s with the creation of the first commercial cell phone, initially used solely for conversation^11^. Today, smartphones allow banking transactions, online shopping, chatting, listening to music, watching videos, and numerous other functions, making them indispensable in people’s daily lives^12^. This “smartphone pandemic” has led to a decline in the sales of analog clocks. Wristwatches are now more commonly regarded as fashion accessories than an item for checking the time. With the dominance of smartphones over analog clocks, questions have arisen about whether CDT was affected^13^.

Since the CDT is used for cognitive screening and considering that dementia is more prevalent in aged populations, it is possible that the dominance of smartphones does not currently impact the use of CDT as a screening tool. This is because many older individuals use smartphones but do not abandon conventional habits, such as checking the time with a wristwatch. In contrast, the younger population is more likely to rely on smartphones and uses traditional watches less frequently^14,15^.

The impact of smartphone use on CDT may become more apparent in 20 to 30 years, as today’s adults become tomorrow’s older people. A recent study involving subjects aged 18–30 years found that participants with self-reported normal cognition scored below the expected range on the CDT. Some of these participants were unable to read the time on a pre-drawn analog clock^16^. This finding raises significant concerns that the CDT may be nearing obsolescence.

Given the limited number of studies addressing the use of the CDT over the years, this study was conducted to investigate whether the prevalence of smartphones over analog clocks has led to a decline in the publication of articles on the CDT. The analysis focused on studies published over the past 30 years (1994 – 2023) and sought to raise new questions regarding the potential impact of smartphone use on the utility of this test.

## METHODS

This bibliometric study targeted articles published in PubMed over the past 30 years. The study complies with the Guideline for Reporting Bibliometric Review of the Biomedical Literature (BIBLIO)^17^.

Inclusion criteria were:

- articles containing the keyword “Clock Drawing Test”;
- published between 1994 and 2023; and
- featured in scientific journals indexed in PubMed.

While there are other databases of significance, the search was limited to PubMed due to its association with the National Library of Medicine and its comprehensive coverage of articles in the fields of Medicine and Health Sciences. Exclusion criteria comprised articles published without prior evaluation by editors and/or reviewers (classified as pre-prints), as well as those that did not use the CDT. Also, articles published only as abstracts were excluded.

The term “Clock Drawing Test” was used to search the database. The reference search was limited to articles published in English, Spanish, or Portuguese. The publications were assessed by two independent researchers. A systematic extraction form was used for each study to collect the following data: year of publication, region in which the study was conducted, and methodological procedure (diagnosis, treatment, review, or letter to the editor). The definition of the period from 1994 to 2023 was not based on any specific event in the field of brain sciences, but rather on the evolution of smartphones in society.

### Statistical analysis

The data were categorized into absolute frequencies and percentages and analyzed on statistical bases. A χ^2^ test was used to compare the number of articles published across three decades (1994–2003; 2004–2013; and 2014–2023), by region (Europe, Asia, Oceania, Africa, North America, Central America, and South America), and according to methodological purposes (diagnosis, treatment, review, or letter to the editor). Cross-tabulations were used for paired comparisons. In all the analyses, significance was set at 5%.

## RESULTS

The PubMed search yielded 1,319 studies. At the full-text review stage, 21 articles were excluded for not meeting the eligibility criteria. The remaining studies were reviewed. Among all the included studies (n=1,298), the number of articles using the CDT has increased significantly over the past 30 years. Table 1 shows the distribution of articles published in each decade.

**Table 1:** Number and percentage of studies published per year.

Characteristic	Year of publication			p-value
	1994–2003	2004–2013	2014–2023	
Number of studies, n	118	405	775	0.001
Increase in the last decade, %	–	243.2	91.3	
Increase in 30 years, %	–	–	556.7	

**Figure 1:**
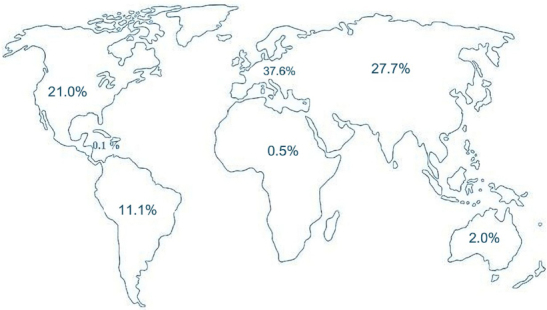
Distribution of Clock Drawing Test studies worldwide.

Notably, most studies using CDT have been conducted in Europe, Asia, and North America. Figure 1 provides a detailed breakdown of the percentage of studies published by region over the past 30 years.

**Figure 2:**
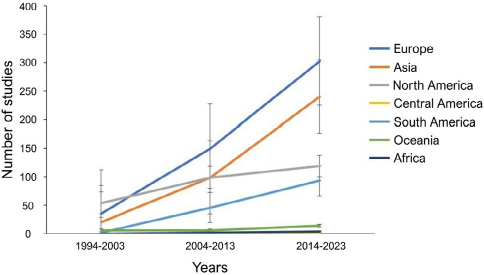
Distribution of studies by region and year.

All regions experienced an increase in the number of publications on CDT. Compared to the total number of studies published in each decade, the most significant increase occurred in Asia (rising from 16.9% in 1994–2003 to 31.3% in 2014–2023), followed by South America (from 2.5% in 1994–2003 to 12.0% in 2014–2023), and Europe (from 29.7% in 1994–2003 to 39.1% in 2014–2023). Figure 2 shows the evolution of publications in each region over the 30-year period.

Most of the publications using the CDT aimed to diagnose people with cognitive impairment (82.2%), followed by treatments studies (11.5%), reviews (5.6%), and letters to the editor (0.7%). Diagnostic studies were more prevalent than other methodological purposes (p=0.001), and this pattern remained consistent across the three decades (p=0.380).

## DISCUSSION

Smartphones have become integral to everyday life, often replacing analog watches^18^. Since drawing an analog watch is the basis of the CDT, this study investigated whether the predominance of smartphones in recent decades may have influenced the number of studies using the CDT. Contrary to the initial hypothesis, the results showed a significant increase in the number of publications with CDT. This increase occurred in all regions, particularly in Europe, Asia, and South America. The findings underscore the continued relevance of the CDT and its importance for health professionals as a diagnostic tool.

More than 50 databases are available for searching studies in the health sciences^19^. Some of these databases are internationally recognized, such as PubMed, EMBASE, CINHAL, Cochrane Library, PsychInfo, and SPORTDiscus, while others are regional databases, such as SciELO, Lilacs, Bireme, and African Index Medicus. Citation reporting databases, such as Scopus and the Web of Science, are also widely used. This study restricted the search to articles published in PubMed for two reasons: first, PubMed provides a broad overview of the existing literature and is more comprehensive than any other database when it comes to the medical sciences; second, searching multiple databases could lead to duplicate inclusion of articles from journals indexed in more than one database. Therefore, readers should be aware that only articles published in journals indexed in PubMed were included.

The data in Table 1 show that the increase in publications on the CDT in PubMed was significantly higher between the first and second decades (243.2% increase) compared to the increase between the second and third decades (increment of 91.3%). When analyzing the total number of studies rather than the percentage increase, the pattern differs: 370 more articles were published in PubMed between 2014–2023 compared to 2004–2013, while 287 additional articles were published between 2004–2013 compared to 1993–2004. These findings confirm that, regardless of whether the percentage of increment or the number of publications is considered, there is no indication of a decrease in the development of studies with CDT.

The results presented in Figure 1 highlight that Europe, Asia, and North America have published the majority of studies on CDT over the last three decades. This trend may reflect higher levels of investment and research funding in these regions compared to the rest of the world. According to UNESCO^20^, the world’s top ten countries in research and development investment are located in North America (the United States invested $581 billion), Asia (China invested $554 billion), and Europe (Germany invested $137 billion).

Although not as prominent in scientific output as Europe, North America, or Asia, research in South America has improved significantly over the years. Science in many South American countries has come a long way since the dark days of dictatorships were extinguished, just a generation ago^21^. The growing number of PhD students can be linked with the rise in CDT-related studies in the region. The scientific output in South America has increased from 2.5% of the total articles with CDT published in PubMed from 1994–2003 to 12.0% of the total studies with CDT published from 2014–2023.

In addition to the increasing number of CDT-related studies in South America, readers should consider the possibility that improved journal indexing over the years has contributed to this growth^22^. It is possible that a significant number of publications existed in South America previously, but the rise in PubMed entries could also reflect an increased number of journals indexed in this database over the years^23^. Further research is needed to investigate this possibility.

Most studies have used the CDT as a tool for cognitive support in clinical diagnosis. The second most common use of the CDT has been to investigate the benefits of interventions on specific aspects of cognitive function. As the world’s population continues to age^24^ and cognitive decline becomes increasingly prevalent in society^25^, it is likely that the increased use of the CDT is associated to these demographic changes over the years. In this context, the use of the CDT is expected to increase, even amidst the technological transition from analog watches to smartphones.

If the CDT remains widely used today, its relevance may change over time. By the age of five, children begin to develop cognitive abilities that allow them to understand clocks and calendars, as well as to identify both recurring cycles (days, weeks, months) and unique events (their birthdays, for example)^26^. However, with the current generation spending much of their time using smartphones^27^, it raises the question of whether the CDT will remain an effective tool for assessing cognitive functions in 50 to 60 years, for a generation that has been immersed in smartphone technology since birth.

Recent studies have highlighted the impact of smartphones and technology on the CDT. Studies involving younger adults indicate that cognitively persevered individuals struggle to draw analog clocks^16,28^. To date, this issue has not significantly affected most publications, as they generally focus on older adults, where technology is present but has not yet fully replaced analog clocks. However, this trend could signal the beginning of the end of the CDT, as today’s younger population will become tomorrow’s older adults. Confirming this premise will require further studies to track the profile of publications using the CDT.

It is acknowledged that the number of studies included in this research was limited, since only 1,298 articles met the eligibility criteria. A rapid search on Google Scholar identified 187,000 CDT citations over the past 30 years. This figure does not necessary mean that there are 187,000 articles published with CDT worldwide, as Google Scholar also includes books, abstracts, and other forms of academic research. Regarding the selected data, we suggest that the 1,298 articles included in this review represent a conservative estimate of only 43.2 articles published per year over the past three decades (far fewer than the total number of studies published across all databases globally). Readers should note that while these data are derived from PubMed, which does not encompass all studies published using CDT, it remains the most prestigious and comprehensive database in the medical sciences.

This study had several limitations. First, the search was restricted to a single database: PubMed. Further studies should be developed to investigate the use of CDT across other databases. Second, the number of journals indexed in PubMed over the years was not accounted for. The increase in the production of CDT studies in PubMed may be related to a possible increase in the number of journals indexed in this database. Third, we did not account for the impact of external events on the development of the articles. For instance, previous studies have identified a negative effect of the COVID-19 pandemic on scientific production^29,30^. Finally, this study did not compare the prevalence of CDT-related studies with those focusing on other cognitive screening tools, such as the Frontal Assessment Battery, Montréal Cognitive Assessment, Verbal Fluency test, or Cambridge Cognition Examination. Such comparisons would help determine whether the CDT is losing ground compared to other important cognitive tests.

In summary, the findings of this study confirm that the CDT has been widely used in clinical practice for cognitive screening of patients with neurodegenerative conditions or dementia. Contrary to the initial hypothesis, the number of studies using the CDT has increased over the past 30 years, suggesting that the transition from analog clocks to smartphones has not yet hindered the use of the CDT. In this context, the topic is intriguing as it opens discussions on future perspectives and potential adjustments in cognitive assessments.

While smartphones emerged long after analog clocks and have had minimal influence on older adults and most publications to date, their influence may be more pronounced in generations raised with smartphones. Further studies are recommended to explore the use of CDT in children and young adults who have grown up with smartphones. Such studies should compare results among young adults who regularly use analog watches *versus* those who do not, and consider varying levels of education to more accurately assess the impact of technology on CDT performance.
